# Estimating HIV transmissions in a large U.S. clinic‐based sample: effects of time and syndemic conditions

**DOI:** 10.1002/jia2.25679

**Published:** 2021-03-16

**Authors:** Satyanand Satyanarayana, Steven A Safren, Brooke G Rogers, Sierra A Bainter, Katerina A Christopoulos, Rob J Fredericksen, William C Mathews, Richard D Moore, Michael J Mugavero, Sonia Napravnik, Adam W Carrico, Matthew J Mimiaga, Kenneth H Mayer, Heidi M Crane

**Affiliations:** ^1^ Department of Psychology University of Miami Coral Gables FL USA; ^2^ The Fenway Institute at Fenway Health Boston MA USA; ^3^ Warren Alpert Medical School of Brown University Providence RI USA; ^4^ Department of Medicine UCSF School of Medicine San Francisco CA USA; ^5^ Department of Medicine University of Washington School of Medicine Seattle WA USA; ^6^ UCSD School of Medicine San Diego CA USA; ^7^ Johns Hopkins University School of Medicine Baltimore MD USA; ^8^ UAB School of Medicine Birmingham AL USA; ^9^ UNC School of Medicine Chapel Hill NC USA; ^10^ Department of Public Health Sciences University of Miami School of Medicine Miami FL USA; ^11^ UCLA Center for LGBTQ Advocacy, Research, and Health (C‐LARAH) Los Angeles CA USA; ^12^ Department of Epidemiology UCLA Fielding School of Public Health Los Angeles CA USA; ^13^ Department of Psychiatry and Biobehavioral Sciences UCLA David Geffen School of Medicine Los Angeles CA USA; ^14^ Massachusetts General Hospital Center for Global Health Boston MA USA; ^15^ Harvard Medical School Boston MA USA

**Keywords:** Cohort studies, HIV prevention, HIV care continuum, viral suppression, treatment, North America

## Abstract

**Introduction:**

Little is known about onward HIV transmissions from people living with HIV (PLWH) in care. Antiretroviral therapy (ART) has increased in potency, and treatment as prevention (TasP) is an important component of ending the epidemic. Syndemic theory has informed modelling of HIV risk but has yet to inform modelling of HIV transmissions.

**Methods:**

Data were from 61,198 primary HIV care visits for 14,261 PLWH receiving care through the Centers for AIDS Research (CFAR) Network of Integrated Clinical Systems (CNICS) at seven United States (U.S.) sites from 2007 to 2017. Patient‐reported outcomes and measures (PROs) of syndemic conditions – depressive symptoms, anxiety symptoms, drug use (opiates, amphetamines, crack/cocaine) and alcohol use – were collected approximately four to six months apart along with sexual behaviours (mean = 4.3 observations). Counts of syndemic conditions, HIV sexual risk group and time in care were modelled to predict estimated HIV transmissions resulting from sexual behaviour and viral suppression status (HIV RNA < 400/mL) using hierarchical linear modelling.

**Results:**

Patients averaged 0.38 estimated HIV transmissions/100 patients/year for all visits with syndemic conditions measured (down from 0.83, first visit). The final multivariate model showed that per 100 patients, each care visit predicted 0.05 fewer estimated transmissions annually (95% confidence interval (CI): 0.03 to 0.06; *p* < 0.0005). Cisgender women, cisgender heterosexual men and cisgender men of undisclosed sexual orientation had, respectively, 0.47 (95% CI: 0.35 to 0.59; *p* < 0.0005), 0.34 (95% CI: 0.20 to 0.49; *p* < 0.0005) and 0.22 (95% CI: 0.09 to 0.35; *p* < 0.005) fewer estimated HIV transmissions/100 patients/year than cisgender men who have sex with men (MSM). Each within‐patient syndemic condition predicted 0.18 estimated transmissions/100 patients/year (95% CI: 0.12 to 0.24; *p* < 0.0005). Each between‐syndemic condition predicted 0.23 estimated HIV transmissions/100 patients/year (95% CI: 0.17 to 0.28; *p* < 0.0005).

**Conclusions:**

Estimated HIV transmissions among PLWH receiving care in well‐resourced U.S. clinical settings varied by HIV sexual risk group and decreased with time in care, highlighting the importance of TasP efforts. Syndemic conditions remained a significant predictor of estimated HIV transmissions notwithstanding the effects of HIV sexual risk group and time in care.

## 1 INTRODUCTION

Recent studies establishing antiretroviral therapy (ART)’s efficacy in preventing transmission of HIV from people living with HIV (PLWH) to their sexual partners have yielded updated per‐act estimates for HIV sexual transmission in the treatment‐as‐prevention (TasP) era [1, 2]. Together with per‐act estimates of HIV transmission when virally unsuppressed [3], these figures illustrate the impact of TasP and undetectable = untransmittable (U = U), wherein consistent adherence to ART by PLWH effectively suppresses their HIV RNA and eliminates their transmission to seronegative partners [4, 5, 6]. As outlined in the UNAIDS 90–90–90 target to end the AIDS epidemic and the United States (U.S.) government’s Ending the HIV Epidemic strategy [7, 8], connecting PLWH to ART and achieving sustained viral suppression are critical goals for improving patient health and preventing the onward transmission of HIV. Recent per‐act estimates of HIV transmission risk highlight the impact of these goals.

Modelling HIV transmissions using per‐act estimates of HIV transmissibility and individually collected behavioural data are rare. Using a three‐country sample (Brazil, Thailand and Zambia) of sexually active PLWH in care, Safren and colleagues in the HIV Prevention Trials Network 063 study estimated the number of transmissions over 15 months using then‐current‐for‐2016 per‐act estimates [9]. That study estimated 3.81 HIV transmissions per 100 patients over 15 months, with between‐country and risk group variations [9]. In the U.S., other studies have based modelling of HIV transmissions on data from U.S. behavioural health surveys and have estimated HIV infections ranging from 0 to 0.2 (for PLWH in care virally suppressed) and 3.8 to 6.1 (for PLWH in care not virally suppressed) transmissions per 100 patients annually [10, 11]. Analyses quantifying the impact of biobehavioural transmission risk behaviour (condomless anal or vaginal sex while virally unsuppressed) among patients in care on estimated HIV transmissions could better inform decision‐making regarding the allocation of treatment and prevention resources to achieve the 90–90–90 goals, particularly given that virologically unsuppressed PLWH in care are estimated to account for 8.5–19.8% of HIV transmissions in the U.S. [11, 12].

Key to this resource allocation will be an understanding of the predictors of estimated HIV transmissions among PLWH in care. Syndemic theory has concerned itself with co‐occurring psychosocial, health and biomedical comorbidities that amplify risk of HIV acquisition [13], and much of syndemic research has focused on analysing the co‐occurrence of psychosocial variables – notably, depressive and/or anxiety symptoms, single‐ or poly‐drug use, alcohol use, childhood sexual abuse (CSA) and intimate partner violence (IPV) – to predict HIV acquisition risk by seronegative individuals [14, 15, 16, 17, 18, 19, 20, 21, 22, 23, 24, 25, 26, 27, 28, 29, 30, 31, 32, 33, 34]. More recent syndemic research has focused on co‐occurring syndemic conditions among PLWH as predictors of HIV transmission risk (e.g. ART nonadherence, uncontrolled viral load or biobehavioural transmission risk behaviour) [35, 36, 37, 38, 39, 40, 41, 42, 43]. Other studies have linked psychosocial variables (notably, depression) and substance use (notably, stimulants), though not their additive effects, to elevated viral load and worse HIV clinical outcomes [9, 52]. To our knowledge, no prior analysis has quantified the impact of co‐occurring syndemic conditions among PLWH on HIV transmission, estimated or otherwise.

Using a large, longitudinal sample of U.S. PLWH in care, we sought to estimate HIV transmissions using recent per‐act estimates and to model the effects of syndemic conditions over and above HIV sexual risk group and time in care on estimated HIV transmissions.

## 2 METHODS

### 2.1 Participants

Participants were 14,261 PLWH receiving care between June 2007 and April 2017 at seven U.S. Centers for AIDS Research (CFAR) Network of Integrated Clinical Systems (CNICS) sites, all of which are well‐resourced, university‐affiliated research medical centres providing the latest treatments in HIV care (see Table 1). Patients 18 years or older were approached at care appointments to participate in CNICS; no reimbursement was offered for participation [53, 54]. Patient‐informed consent was obtained during initial enrolment in CNICS. Procedures involving human participants were in accordance with institutional review boards at the CNICS‐affiliated universities and with the 1964 Helsinki Declaration and its later amendments or comparable ethical standards.

**Table 1:** CNICS sites included in analyses

CNICS site	U.S. City, State
University of Alabama at Birmingham	Birmingham, Alabama
University of California San Francisco	San Francisco, California
University of Washington	Seattle, Washington
University of California San Diego	San Diego, California
Fenway Health/Harvard University	Boston, Massachusetts
University of North Carolina at Chapel Hill	Chapel Hill, North Carolina
Johns Hopkins University	Baltimore, Maryland

CNICS, Centers for AIDS Research (CFAR) Network of Integrated Clinical Systems.

### 2.2 Data sources

The CNICS data repository integrates data from electronic health records and institutional data sources with data collected upon study enrolment [53, 54]. Self‐administered patient‐reported outcomes and measures (PROs) are collected at least four to six months apart as part of clinical care via touch‐screen tablets or computers [53, 54].

### 2.3 Procedures and measures

#### 2.3.1 Measures

PROs include: past‐two‐week depressive symptoms measured by the Patient Health Questionnaire–9 (PHQ–9) [55]; past‐month panic symptoms measured by five items from the Brief Patient Health Questionnaire (PHQ–5) [56]; past‐three‐month use of amphetamines, illicit opioids and crack/cocaine measured using the Alcohol, Smoking and Substance Involvement Screening Test (ASSIST) [57]; past‐year alcohol consumption measured using versions of the Alcohol Use Disorders Identification Test (AUDIT) or the first three questions of the AUDIT (AUDIT‐C) [58]; and past‐six‐month number of sexual partners and frequency of sex and condom use measured by a modified version of the HIV Risk Assessment for Positives (HRAP) [59].

#### 2.3.2 HIV risk group classification

Patients were initially classified into HIV sexual risk groups based on sex, gender identity and self‐identified sexual orientation where available. After reviewing self‐reported lifetime sexual behaviours of self‐identified cisgender men who have sex with men (MSM) in the sample, a lifetime history of anal sex and no lifetime history of vaginal sex were used as criteria to classify additional cisgender men with undisclosed sexual orientation as MSM. (Most remaining cisgender men of undisclosed sexual orientation reported histories of vaginal and anal sex (1741), whereas smaller numbers reported vaginal sex only (630) or no sex (578)). This overall process yielded 2,239 cisgender women (15.7%), 163 transgender women (1.1%), 1,183 cisgender heterosexual men (8.3%), 7,727 cisgender MSM (54.2%) and 2,949 cisgender men of undisclosed sexual orientation (20.7%).

#### 2.3.3 Imputation of missing PROs

Rates of missingness for complete responses on the PHQ–9, the PHQ–5, the ASSIST, the AUDIT/AUDIT‐C and the HRAP during clinic visits when PROs were administered are included in Table 2. Multilevel multiple imputation using the fully conditional specification algorithm in Blimp (versions 2.1.3 or greater) was used to generate 100 complete data sets [60, 61, 62]. Demographic variables, HIV‐related treatment variables and PRO responses were included in imputation models at each imputation stage to minimize potential for bias [63]. All PROs, with the exception of sexual behaviours, were imputed in the first imputation stage; due to skip patterns in the HRAP questionnaire, imputation of missing sexual behaviour data took place in successive stages [64]. All subsequent data analyses were completed across all 100 imputed data sets.

**Table 2:** Missingness of non‐imputed patient‐reported outcomes and measures (PROs)

PROs	Number of complete responses	Number of incomplete/missing responses	Percentage of missingness
PHQ–9	53,574	7,624	12.5%
PHQ–5	55,850	5,348	8.7%
ASSIST			
Cocaine/crack	53,924	7,274	11.9%
Illicit opioids	50,530	10,668	17.4%
Methamphetamine	53,927	7,271	11.9%
AUDIT/AUDIT–C	54,450	6,748	11.0%
Sexual behaviours^a^			
Anal sex, seronegative partner	46,301	14,897	24.3%
Condom use	47,719	13,479	22.0%
Anal sex: serostatus unknown	49,507	11,691	19.1%
Condom use	48,061	13,137	21.5%
Vaginal sex: seronegative partner	48,055	13,143	21.5%
Condom use	48,387	12,811	20.9%
Vaginal sex: serostatus unknown	49,328	11,870	19.4%
Condom use	49,110	12,088	19.8%

PRO, patient‐reported outcomes and measures.

^a^ High missing data rates for sexual behaviours recorded using the HIV Risk Assessment for Positives (HRAP) were due in part to transitions to a new sexual risk measure in late 2016/early 2017 (date varied by site, median date 9/2016).

#### 2.3.4 Quantification of sexual behaviours

In four separate questions, the HRAP queried past‐six‐month frequency of sexual behaviour by type of sex (anal and vaginal) and by serostatus of partner (seronegative and serostatus‐unknown). Responses were converted to count values: *a few times each week* as 78 (three per week over 6 months); *a few times each month* as 18 (three per month for six months); *a few times or less* as 1.5; and *never* as zero. The HRAP also queried frequency of condom use for each type of sexual behaviour; responses were converted to proportions as follows: *all of the time* as 100%; *most of the time* as 75 %; *some of the time* as 25% and *never* as 0%.

#### 2.3.5 Estimation of HIV transmissions from sexual behaviours

Procedures to derive estimates of HIV transmissions were similar to those used in the HPTN063 study but with updated per‐act estimates [9]. Condom‐use proportions were multiplied by appropriate sex‐act counts to calculate totals for eight separate sexual behaviours based on sex act, condom use and partner serostatus. Each total was multiplied by the appropriate per‐act estimate of HIV transmission risk based on viral suppression, sex act and condom use as indicated in Table 3 [1, 2, 3]. Where unavailable in the literature, per‐act estimates for sex with condoms were calculated by reducing per‐act estimates of the corresponding condomless sex acts by 80% [3, 65]. Because the HRAP did not query whether anal sex was insertive or receptive, per‐act estimates for insertive and receptive anal sex were averaged for all groups except cisgender heterosexual men and cisgender women (Table 3).

**Table 3:** Per‐sex‐act percentage estimates of HIV transmission risk

Sex act	Virally suppressed	Cisgender heterosexual men (% per act)		Cisgender MSM (% per act)		Cisgender men of undisclosed sexual orientation (% per act)		Cisgender women (% per act)		Transgender women (% per act)	
		Condom use		Condom use		Condom use		Condom use		Condom use	
		Yes	No	Yes	No	Yes	No	Yes	No	Yes	No
Vaginal	Yes	0.00078^a^	0.0039^b^	0.00078^a^	0.0039^b^	0.00078^a^	0.0039^b^	0.00078^a^	0.0039^b^	0.00078^a^	0.0039^b^
Vaginal	No	0.016^c^	0.081^c^	0.016^c^	0.081^c^	0.016^c^	0.081^c^	0.008^c^	0.042^c^	0.016^c^	0.081^c^
Anal	Yes	0.00088^a^	0.0044^b^	0.00088^a^	0.0044^b^	0.00088^a^	0.0044^b^	0.00088^a^	0.0044^b^	0.00088^a^	0.0044^b^
Anal	No	0.334^d^	1.67^e^	0.181^f^	0.905^g^	0.181^f^	0.905^g^	0.028^d^	0.14^e^	0.181^f^	0.905^g^

^a^ Per‐act estimates drawn from Supervie and Breban and downwardly adjusted by 80%, consistent with Patel and colleagues and the findings of Weller and Davis‐Beaty[1, 3, 65]

^b^ per‐act estimates drawn from Supervie and Breban[1]

^c^ per‐act estimates drawn from Patel and colleagues[3]

^d^ per‐act estimates drawn from Baggaley and colleagues and downwardly adjusted by 80% consistent with Patel and colleagues and the findings of Weller and Davis‐Beaty[2, 3, 66]

^e^ per‐act estimates drawn from Baggaley and colleagues[2]

^f^ average of insertive and receptive anal sex per‐act estimates drawn from Baggaley and colleagues and downwardly adjusted by 80% consistent with Patel and colleagues and the findings of Weller and Davis‐Beaty[2, 3, 48]

^g^ average of insertive and receptive anal sex per‐act estimates drawn from Baggaley and colleagues [2].

To account for different viral load thresholds across time and sites, viral suppression was set at HIV RNA < 400/mL, with viral suppression status extracted from patients’ electronic health records. Estimated transmissions to serostatus‐unknown partners were reduced by 25% based upon an assumption that one in four such partners were seropositive. The resulting eight estimates were summed to calculate totals of estimated HIV transmissions per patient per six‐month observation period, which were then doubled to represent annualized estimates. For ease of interpretation, annualized estimates were multiplied by 100 to reflect the number of estimated HIV transmissions per 100 patients annually. Sensitivity analyses were conducted to account for 10% preexposure prophylaxis (PrEP) adherence among seronegative partners (PrEP), 50% seropositivity among serostatus‐unknown partners and 0% transmission risk for virally suppressed patients using condoms.

#### 2.3.6 Syndemic conditions

Four syndemic conditions were assessed from PROs: (1) clinically elevated depressive symptoms (≥5 on the PHQ–9) [55]; (2) any anxiety symptoms rated on the PHQ–5 (experiencing an anxiety attack in the previous four weeks); (3) screening positive for a substance use disorder (≥4 on the ASSIST for cocaine/crack, illicit opioids or amphetamines) [67] and 4) screening positive for heavy drinking/active alcohol use disorders (≥ 4 for cisgender men and transgender women or ≥3 for cisgender women) on the AUDIT‐C or the first three questions of the AUDIT [58].

Between‐person syndemic scores (mean number of syndemic conditions) were calculated for each patient for between‐patient comparisons. Within‐person syndemic scores (observed number of syndemic conditions minus between‐person syndemic score) were calculated for each observation to model individual variations in patients’ syndemic conditions over time.

#### 2.3.7 Time in care

Because patients were seen on different dates at different stages of treatment and for differing periods of time, time in care was measured by number of visits since PRO collection began in their respective clinic.

### 2.4 Data analysis

Hierarchical linear modelling using restricted maximum likelihood estimation was employed for analyses across 100 imputed data sets using R version 4.0.0 and the lme4, lmerTest and mitml packages [68, 69, 70, 71]. Standard errors were computed using Satterthwaite’s approximation.

A series of models was fitted to determine the effect of syndemic conditions on estimated HIV transmissions; control variables – time in care and HIV sexual risk group – were limited to those most closely hypothesized to be related to estimated HIV transmissions over and above syndemic conditions. Model 1, an intercept‐only model, was run with estimated HIV transmissions as the outcome to determine the proportion of variance attributable to between‐person differences in estimated HIV transmissions. Model 2 added the fixed effects of time in care. Model 3 added the fixed effects of HIV sexual risk group. Model 4 added the fixed effects of within‐person syndemic scores, whereas Model 5 added the fixed effects of between‐person syndemic scores.

## 3 RESULTS

Tables 4 and 5 contain descriptive statistics of relevant data for patients at first PRO visit (Table 4) and for all PRO visits (Table 5). Across all visits, depressive symptoms were most prevalent (46.4%) followed by anxiety symptoms (26.9% all visits), then alcohol consumption (23.9% all visits) and then illicit drug use (14.9% all visits) (Table 5). At first PRO visit, rates of viral suppression were lower, and estimated HIV transmissions and syndemic condition prevalence higher, than for subsequent visits (Tables 4, 5). Across all visits, patients had 0.38 estimated HIV transmissions per 100 patients annually (SD = 4.23) (down from 0.83 (SD = 6.66) at first PRO visit), with transgender women having the greatest number and cisgender women the least (Tables 4, 5). Table 6 provides descriptive statistics of estimated HIV transmissions per 100 patients annually by syndemic condition count and HIV sexual risk group for all visits.

**Table 4:** Sample characteristics at first PRO visit

Variable	Risk group					
	All (N = 14,261)	Cisgender heterosexual Men (n = 1183)	Cisgender MSM (n = 7727)	Cisgender men, undisclosed Sex. Orient. (n = 2949)	Cisgender women (n = 2,239)	Transgender women (n = 163)
Age: M (SD)	43.7 (11.0)	48.5 (10.5)	42.1 (10.8)	45.0 (10.9)	45.4 (10.8)	41.2 (10.6)
Race: (%)						
White	8,234 (57.7)	437 (36.9)	5,268 (68.2)	1,723 (58.4)	743 (33.2)	63 (38.6)
Black	4,696 (32.9)	682 (57.7)	1,621 (21.0)	970 (32.9)	1,355 (60.5)	68 (41.7)
Native American	125 (0.9)	8 (0.7)	60 (0.8)	28 (0.9)	26 (1.2)	3 (1.8)
Asian/PacificIslander	359 (2.5)	14 (1.2)	260 (3.3)	49 (1.7)	29 (1.3)	7 (4.3)
Multiracial	87 (0.6)	0 (0.0)	66 (0.9)	16 (0.5)	3 (0.1)	2 (1.2)
Other/unknown	760 (5.3)	42 (3.6)	452 (5.8)	163 (5.5)	83 (3.7)	20 (12.3)
Hispanic/Latinx: (%)	2,018 (14.2)	135 (11.4)	1,221 (15.8)	399 (13.5)	212 (9.5)	51 (31.3)
Syndemic condition: (%)^a^						
Depressive Sxs	7,228 (50.7)	503 (42.5)	3,882 (50.2)	1,538 (52.2)	1,202 (53.7)	102 (62.6)
Anxiety Sxs	4,097 (28.7)	206 (17.4)	2,297 (29.7)	875 (29.7)	644 (28.8)	75 (46.0)
Illicit Drug Use	2,607 (18.3)	188 (15.9)	1,328 (17.2)	725 (24.6)	330 (14.7)	35 (21.5)
Alcohol consumption	3,957 (27.7)	267 (22.6)	2,330 (30.2)	839 (28.5)	487 (21.8)	34 (20.8)
No. of syndemic conditions: M (SD)^a^	1.25 (1.08)	0.98 (1.02)	1.27 (1.06)	1.35 (1.12)	1.19 (1.07)	1.51 (1.09)
Virally suppressed: (%)^b^	10,760 (75.5)	944 (79.8)	5,868 (75.9)	2148 (72.8)	1,675 (74.8)	125 (76.7)
Est. No. of HIV Trans. per 100 pts. per year: M (SD)^a^	0.83 (6.66)	0.34 (6.23)	1.20 (8.11)	0.59 (4.81)	0.10 (0.79)	1.30 (5.94)

M, mean; PRO, patient‐reported outcomes and measures; pts, patients; SD, standard deviation; Sex. Orient., sexual orientation; Sxs, symptoms; Trans., transmissions; MSM, men who have sex with men.

^a^ Values from imputed data sets

^b^ viral suppression defined as <400 RNA/mL.

**Table 5:** Sample characteristics, All PRO visits

Variable	Risk group					
	All (N = 61,198)	Cisgender heterosexual men (n = 5,983)	Cisgender MSM (n = 37,019)	Cisgender men, undisclosed Sex. Orient. (n = 7,782)	Cisgender women (n = 9814)	Transgender women (n = 600)
Age: M (SD)	46.1 (10.8)	49.6 (10.1)	45.1 (10.8)	46.4 (10.7)	47.3 (10.5)	43.4 (9.8)
Race: (%)						
White	36,444 (60.0)	2,541 (42.5)	25,697 (69.4)	4,593 (59.0)	3,326 (33.9)	287 (47.8)
Black	20,504 (33.5)	3,153 (52.7)	8,442 (22.8)	2,658 (34.2)	6,017 (61.3)	234 (39.0)
Native American	463 (0.8)	36 (0.6)	257 (0.7)	52 (0.7)	105 (1.1)	13 (2.2)
Asian/Pacific Islander	1,287 (2.1)	69 (1.2)	979 (2.6)	103 (1.3)	117 (1.2)	19 (3.2)
Multiracial	300 (0.5)	0 (0.0)	231 (0.6)	40 (0.5)	26 (0.3)	3 (0.5)
Other/unknown	2200 (3.6)	184 (3.1)	1,413 (3.8)	336 (4.3)	223 (2.3)	44 (7.3)
Hispanic/Latinx: (%)	8,699 (14.2)	850 (14.2)	5,753 (15.5)	1,008 (13.0)	907 (9.2)	181 (30.2)
No. of clinic visits: M (SD)	4.29 (3.82)	5.06 (4.29)	4.79 (3.93)	2.64 (2.35)	4.38 (4.15)	3.68 (3.32)
No. of days between visits: M (SD)	359.2 (293.3)	353.1 (315.9)	356.7 (271.5)	360.4 (304.1)	363.2 (337.8)	465.5 (408.8)
Syndemic condition: (%)^a^						
Depressive Sxs	28,382 (46.4)	2,380 (39.8)	16,915 (45.7)	3,911 (50.3)	4,815 (49.1)	361 (60.2)
Anxiety Sxs	16,484 (26.9)	1,145 (19.1)	9,985 (27.0)	2,301 (29.6)	2,799 (28.5)	255 (42.5)
Illicit drug use	9,129 (14.9)	833 (13.9)	5,258 (14.2)	1,714 (23.1)	1,218 (12.4)	106 (17.7)
Alcohol consumption	14,626 (23.9)	1,188 (19.9)	9,366 (25.3)	2,024 (26.1)	1,918 (19.5)	130 (21.7)
No. of syndemic conditions: M (SD)^a^	1.12 (1.05)	0.93 (1.02)	1.12 (1.03)	1.28 (1.11)	1.10 (1.05)	1.42 (1.08)
Virally suppressed: (%)^b^	52,077 (85.1)	5,275 (88.2)	31,983 (86.4)	6,219 (79.9)	8,084 (82.4)	516 (86.0)
Est. No. of HIV Trans. per 100 pts per year: M (SD)^a^	0.38 (4.23)	0.14 (2.96)	0.50 (4.90)	0.39 (4.23)	0.07 (0.52)	0.68 (4.21)
No. of patient years of sexual behaviour	27,462.2	2656.3	16,584.7	3572.2	4,378.2	270.8

M, mean; MSM, men who have sex with men; pts, patients; PRO, patient‐reported outcomes and measures; SD, standard deviation; Sex. Orient., sexual orientation; Sxs, symptoms; Trans., transmissions.

^a^ Values from imputed data sets

^b^ viral suppression defined as <400 RNA/mL.

**Table 6:** Estimated HIV Transmissions by syndemic condition count and sexual risk group

No. of Syndemic conditions^a^	Estimated HIV transmissions per 100 patients per year: M (SD)^a^					
	All	Cisgender heterosexual men	Cisgender MSM	Cisgender men, undisclosed Sex. Orient.	Cisgender women	Transgender Women
0	0.19 (2.70)	0.05 (0.49)	0.25 (3.31)	0.25 (2.62)	0.05 (0.31)	0.15 (1.34)
1	0.30 (3.39)	0.12 (1.09)	0.37 (3.82)	0.31 (4.04)	0.06 (0.40)	0.91 (5.06)
2	0.53 (5.22)	0.14 (2.42)	0.69 (5.99)	0.57 (5.84)	0.07 (0.38)	0.35 (2.81)
3	0.87 (6.66)	0.42 (6.40)	1.18 (7.93)	0.52 (4.07)	0.15 (1.02)	1.51 (6.54)
4	1.32 (8.72)	1.62 (15.98)	1.83 (9.61)	0.80 (4.08)	0.25 (1.87)	2.21 (6.68)

M, mean; MSM, men who have sex with men; SD, standard deviation; Sex. Orient., sexual orientation.

^a^ Values from imputed data sets.

Estimates from the five iterative models are displayed in Table 7. Model 1, the intercept‐only model, yielded an intraclass correlation (ICC) of .086, indicating 8.6% of variability in estimated HIV transmissions was attributable to individual‐level differences. Model 2 showed that each subsequent visit predicted 0.05 fewer estimated HIV transmissions per 100 patients annually, an effect that remained the same in all subsequent models. Model 3 showed that, controlling for the effects of time, cisgender women, cisgender heterosexual men and cisgender men of undisclosed sexual orientation had, respectively, 0.47, 0.39 and 0.20 fewer estimated HIV transmissions per 100 patients annually relative to cisgender MSM. Model 4 revealed that each within‐person syndemic condition predicted 0.18 estimated HIV transmissions per 100 patients annually, controlling for time in care and risk group, whereas the effects of the risk group remained largely unchanged from Model 3.

**Table 7:** Models of predicting estimated HIV transmissions over time

	Model 1^a^ (Intercept‐only)	Model 2^b^ (Time in care)	Model 3^c^ (Risk Group)	Model 4^d^ (Within‐Person Syndemic Conditions)	Model 5^e^ (Between‐Person Syndemic Conditions)
**Fixed effects**					
Intercept: β_00_ (SE)	0.42 (0.023)***	0.61 (0.032)***	0.75 (0.039)***	0.74 (0.039)***	0.47 (0.048)***
95% CI	0.38 to 0.47	0.55 to 0.67	0.67 to 0.82	0.67 to 0.82	0.37 to 0.56
Time in care: β_10_ (SE)		−0.05 (0.006)***	−0.05 (0.006)***	−0.05 (0.006)***	−0.05 (0.006)***
95% CI		−0.06 to −0.04	−0.06 to −0.04	−0.06 to −0.04	−0.06 to −0.03
Risk group^#^:					
Cisgender heterosexual men: β_21_ (SE)			−0.39 (0.076)***	−0.39 (0.076)***	−0.34 (0.075)***
95% CI			−0.54 to −0.24	−0.54 to −0.24	−0.49 to –0.20
Cisgender men, Und. Sex. Orient.: β_22_ (SE)			−0.20 (0.066)**	−0.20 (0.066)**	−0.22 (0.066)**
95% CI			−0.33 to –0.07	−0.32 to −0.07	−0.35 to –0.09
Cisgender women: β_23_ (SE)			−0.47 (0.061)***	−0.47 (0.061)***	−0.47 (0.061)***
95% CI			−0.59 to −0.35	−0.59 to −0.35	−0.59 to −0.35
Transgender women: β_24_ (SE)			0.12 (0.233)	0.12 (0.233)	0.06 (0.232)
95% CI			−0.34 to 0.57	−0.34 to 0.57	−0.39 to 0.52
Within‐person Syndemic Conditions: β_30_ (SE)				0.18 (0.031)***	0.18 (0.031)***
95% CI				0.12 to 0.24	0.12 to 0.24
Between‐person syndemic conditions: β_40_ (SE)					0.23 (0.027)***
95% CI					0.17 to 0.28
Random effects					
Intercept:$\sigma_{\text{u0}}^{2}$	1.59	1.56	1.52	1.53	1.49
Residual:$\sigma_{e}^{2}$	16.50	16.50	16.50	16.48	16.48

95% CI, 95% confidence interval; SE, standard error; Und. Sex. Orient., undisclosed sexual orientation.

*p < .05; **p < .005; ***p < .0005; ^#^Cisgender men who have sex with men as referent group.

^a^ Model 1: transmission_ti_ = β_00_ + u_0i_ + e_ti_

^b^ Model 2: transmission_ti_ = β_00_ + β_10_ time + u_0i_ + e_ti_

^c^ Model 3: transmission_ti_ = β_00_ + β_10_ time + β_20_ riskgroup + u_0i_ + e_ti_

^d^ Model 4: transmission_ti_ = β_00_ + β_10_ time + β_20_ riskgroup + β_30_ withinperson_ti_ + u_0i_ + eti

^e^ Model 5: transmission_ti_ = β_00_ + β_10_ time + β_20_ riskgroup + β_30_ withinperson_ti_ + β_40_ betweenperson_ti_ + u_0i_ + e_ti._

Model 5 included the effects of between‐person syndemic conditions with the prior set of predictors. Controlling for time in care and risk group, each between‐person syndemic condition predicted an additional 0.23 estimated HIV transmissions per 100 patients annually, whereas as in Model 4, each within‐person syndemic condition predicted 0.18 estimated HIV transmissions per 100 patients annually. Cisgender women, cisgender heterosexual men and cisgender men of undisclosed sexual orientation had, respectively, 0.47, 0.34 and 0.22 fewer estimated HIV transmissions per 100 patients annually compared to cisgender MSM. Sensitivity analyses assuming scenarios that 10% of seronegative partners were PrEP‐adherent, 50% seropositivity among serostatus‐unknown partners and 0% transmission risk when virally suppressed and using condoms did not alter the general pattern of estimates (see Table S1).

## 4 DISCUSSION

To our knowledge, our modelling study is the first to demonstrate the significant effects of syndemic conditions, HIV sexual risk group and time in care on estimated HIV transmissions using a large sample of PLWH in clinical care. We found syndemic conditions – both within‐patient and between‐patient – predicted increased estimated HIV transmissions, whereas each additional patient visit predicted smaller but significant decreases in estimated HIV transmissions. Our modelling also predicted fewer estimated HIV transmissions for cisgender women, cisgender heterosexual men and cisgender men of undisclosed sexual orientation compared to cisgender MSM; transgender women’s point estimate was higher than cisgender MSM’s, but the difference between groups was not significant, likely due to the small number of transgender women in the sample.

While the effects of syndemic conditions on estimated HIV transmissions were modest, they should be contextualized within the treatment settings where they arose and alongside the significant effects of time in care. CNICS clinics are resource‐rich, research medical centres where viral suppression rates approached 90% in recent years [72], and where patients have access to a variety of case management services and HIV prevention messaging. They also boast retention‐in‐care (RIC) outcomes nearly a third greater than the U.S. clinic average [73]. Within this context and the availability of more potent and administrable ART regimens in recent years, syndemic conditions still predicted estimated HIV transmissions, even after accounting for time in care and attendant increases in viral suppression; the effect of syndemic conditions on estimated HIV transmissions might potentially be greater in more resource‐constrained settings. Moreover, the longitudinal assessment of within‐patient changes in syndemic conditions was predictive of transmission risk beyond between‐patient differences, suggesting the value of monitoring individual‐level syndemic trajectories over time with tools like PROs embedded in routine care. Such monitoring could result in additional RIC efforts towards vulnerable patients as well as referrals to alleviate distress associated with syndemic conditions as they arise, with the critical benefit of also diminishing potential onward HIV transmission. Increased efforts accordingly must be made in HIV care settings to consistently screen for syndemic conditions [74, 75].

Our study adds to growing literature on syndemic conditions and transmission risk behaviour among PLWH. Prior studies of syndemic conditions among PLWH showed additive associations with increased odds of ART non‐adherence [36, 37, 38, 39, 40, 43], virological non‐suppression [38, 40, 43] and condomless sex with serodiscordant partners while virally unsuppressed [42, 43]. However, these studies have generally treated the outcomes of interest as dichotomous outcomes: ART adherent versus non‐adherent [36, 39, 40, 43]; virally suppressed versus unsuppressed [40, 43]; and the presence or absence of condomless sex while virally unsuppressed [42, 43]. Our study builds upon these past findings by quantifying estimated HIV transmissions longitudinally associated with co‐occurring syndemic conditions among a diversity of risk groups of PLWH in care.

Our study also adds to the literature regarding the prevalence of syndemic conditions among PLWH in care. Prevalence in our sample for clinically significant depressive symptoms (46.4% all visits), anxiety symptoms (26.9% all visits), current (3‐month) illicit drug use (not including marijuana) (14.9% all visits) and risky drinking (23.9% all visits) are generally consistent with estimates of prevalence for these conditions among PLWH, acknowledging that different studies have used different measures and cut‐points with particular subsets of patients [76, 77]. Patients in all HIV risk groups averaged a greater number of syndemic conditions at first PRO visit compared to all PRO visits. Transgender women in our sample averaged the greatest number of syndemic conditions among HIV sexual risk groups, consistent with other literature highlighting their elevated syndemic burden and the need for affirming and supportive interventions for this population [66].

The effects of time in care on estimated HIV transmissions, whereas smaller, were nevertheless significant. At a cohort level, the increase in viral suppression over time has been attributed, in part, to the advent of integrase strand transfer inhibitors (ISTIs). At the individual level, our finding raises questions for future study as to whether the effects of time in care are a function of retention in care and resultant viral suppression, of decreased sexual activity over time (or once in care), and/or of increased prevention behaviours resulting from clinic prevention messaging.

This study comes with several limitations. First, our study focused on PLWH in care and therefore excluded PLWH who never received, or were not retained in, care. Second, no actual HIV transmissions were measured as part of this modelling study. Third, because this was an observational study, causality cannot be inferred despite the longitudinal design. Fourth, certain syndemic conditions in the HIV syndemic literature – IPV, CSA and violence exposure – were not measured via PRO during the study period and therefore not modelled. Fifth, our measure of sexual transmission risk behaviour did not account for PrEP use by seronegative partners nor serostatus of serostatus‐unknown partners, though sensitivity analyses accounting for 10% PrEP adherence among seronegative partners and the unlikely scenario of 50% of serostatus‐unknown partners being seropositive did not alter our pattern of estimates. Sixth, assumptions were made regarding the quantification of qualitative responses on the HRAP. Seventh, our modelling assumed small but non‐zero per‐act estimates of HIV transmission risk and an 80% reduction in transmission risk when condoms were used, based on estimates in the literature; however, a sensitivity analysis assuming 0% risk of transmission when virally suppressed and using condoms did not alter the pattern of results. Eighth, our study averaged per‐act estimates for insertive and receptive anal sex for anal sex behaviours by cisgender MSM, cisgender men of undisclosed sexual orientation and transgender women due to the limitations of the HRAP questionnaire, which may have failed to account for other HIV risk mitigation strategies (e.g. increased condom use during insertive anal sex or increased rates of receptive anal sex generally) [78]. Ninth, the use of a viral suppression threshold of HIV RNA < 400/mL is higher than the typical threshold of HIV RNA < 200/mL and suggests our estimates of HIV transmissions from this sample are conservative.

A future direction of study is the measurement of structural syndemic conditions (e.g. housing instability, criminal justice involvement, poverty [43, 79]) to understand how structural barriers influence individual transmission risk. Measurement of structural syndemic conditions would bolster a case for testing interaction effects among the syndemic conditions to enable an understanding of how variables at multiple levels exacerbate HIV health outcomes [80, 81]. Our study’s focus on individual‐level syndemic conditions measured at clinic visits made the case for testing interaction effects less persuasive. Particularly in the context of clinical settings where psychosocial interventions for depression, anxiety and drug and alcohol abuse are provided to patients individually, judgements of both clinician and patient are critical in guiding decisions of which patient‐level problems to address and how and when to address them. As such, testing for interactions between individual‐level syndemic conditions, with an eye to potentially deploying single‐component interventions, is of arguably less importance, particularly as effective transdiagnostic treatments of mood, anxiety and substance use disorders emerge to treat multiple syndemic conditions [82].

## 5 CONCLUSIONS

This study quantifies estimated HIV transmissions among PLWH in care at well‐resourced U.S. clinics and predicts the effects of syndemic conditions on HIV transmissions over and above time in care and HIV sexual risk group. Compared to the effects of the HIV sexual risk group or cumulative time in care, the impact of syndemic conditions on estimated HIV transmissions was modest but persistent over time. The effect of syndemic conditions on HIV transmissions might be more dramatic in resource‐constrained settings or among PLWH not in care. However, clinics like the CNICS sites, which provide potent ART regimens, strong case management services and psychosocial referrals, are providing patient care leading to fewer estimated HIV transmissions over time. Our findings strongly suggest the need for increased allocation of resources towards linkage‐to‐care and retention‐in‐care initiatives to stem the tide of the HIV epidemic.

## Competing Interests

Dr. Safren receives royalties from Oxford University Press, Springer/Humana Press and Guilford Publications for books on cognitive behavioural therapy.

## Authors’ contributions

SS and SAS devised the study design in consultation with MJM2, HMC and AWC. SS performed statistical analyses for the study with assistance and advising from SAB and BGR. SS wrote the first draft of the manuscript with assistance from SAS. KAC, RJF, WCM, RDM, MJM1, SN, KHM and HMC were site investigators overseeing the collection of CNICS data. All authors reviewed, approved and contributed to editing the final manuscript.

## Supporting information


**Table S1.** Sensitivity analyses of final model predicting estimated HIV transmissions over time
