# Supplementary material for: Estimating HIV transmissions in a large U.S. clinic‐based sample: effects of time and syndemic conditions
Source: J Int AIDS Soc. 2021 Mar 16;24(3):e25679. doi: 10.1002/jia2.25679 (PMC7962793; doi:10.1002/jia2.25679)
Supplement: Supplementary file 1 — Table S1. Sensitivity analyses of final model predicting estimated HIV transmissions over time [file JIA2-24-e25679-s001.docx]

**Supplementary Tables**

**Table S1. Sensitivity Analyses of Final Model Predicting Estimated HIV Transmissions Over Time**

|  | Scenario 1:  10% PrEP Adherence Among Seronegative Partners | Scenario 2:  50% of Serostatus-Unknown Partners are Seropositive | Scenario 3:  0% Transmission Risk for Condom Use While Virally Suppressed |
| --- | --- | --- | --- |
| **Fixed Effects** |  |  |  |
| Intercept: $\text{β}_{00} \left( SE \right)$ | 0.43 (0.044)******* | 0.43 (0.046)*** | 0.46 (0.048)*** |
| *95% CI* | *0.34 to 0.52* | *0.35 to 0.52* | *0.37 to 0.56* |
| Time in Care: $\text{β}_{10}(SE)$ | −0.04 (0.005)*** | −0.04 (0.005)*** | −0.05 (0.006)*** |
| *95% CI* | *−0.05 to −0.03* | *−0.05 to −0.03* | *−0.06 to −0.03* |
| Risk Group^#^: |  |  |  |
| Cisgender Heterosexual Men: $\text{β}_{21} (SE)$ | −0.32 (0.069)*** | −0.31 (0.071)*** | −0.35 (0.075)*** |
| *95% CI* | *−0.46 to –0.18* | *−0.45 to −0.17* | *−0.50 to −0.20* |
| Cisgender Men, Und. Sex. Orient.: $\text{β}_{22}$ (*SE*) | −0.21 (0.060)** | −0.19 (0.062)** | −0.22 (0.066)** |
| *95% CI* | *−0.33 to –0.09* | *−0.32 to −0.07* | *−0.35 to −0.09* |
| Cisgender Women: $\text{β}_{23} (SE)$ | −0.43 (0.056)******* | −0.42 (0.057)*** | −0.47 (0.061)*** |
| *95% CI* | *−0.54 to −0.32* | *−0.54 to −0.31* | *−0.59 to −0.35* |
| Transgender Women: $\text{β}_{24} (SE)$ | 0.05 (0.213) | 0.06 (0.220) | 0.06 (0.232) |
| *95% CI* | *−0.36 to 0.47* | *−0.37 to 0.49* | *−0.40 to 0.51* |
| Within-Person Syndemic Conditions: $\text{β}_{30}$ (*SE*) | 0.17 (0.029)******* | 0.16 (0.030)*** | 0.18 (0.031)*** |
| *95% CI* | *0.11 to 0.23* | *0.11 to 0.22* | *0.12 to 0.24* |
| Between-Person Syndemic Conditions: $\text{β}_{40}$ (*SE*) | 0.21 (0.025)******* | 0.20 (0.025)*** | 0.23 (0.027)*** |
| *95% CI* | *0.16 to 0.26* | *0.15 to 0.25* | *0.17 to 0.28* |
| **Random Effects** |  |  |  |
| Intercept: $\text{σ}_{\text{u0}}^{2}$ | 1.26 | 1.31 | 1.48 |
| Residual: $\text{σ}_{\text{e}}^{2}\text{ }$ | 13.99 | 14.49 | 16.49 |

*Note*. *SE* = standard error; 95% CI = 95% confidence interval; Und. Sex. Orient. = undisclosed sexual orientation.
******p* < .05; *******p* < .005; ********p* < .0005**;** ^#^Cisgender men who have sex with men as referent group.
